# 

**Published:** 1888-08

**Authors:** 


### The Independent Practitioner—Vol. IX.

### PUBLISHED BY DENTISTS FOR DENTISTS.

### Prospectus.

With the present number THE INDEPENDENT PRACTITIONER assumes a more important role, as the organ of the International Dental Journal Association. The promises of the New York Dental Journal Association of making volume IX better than any former volumes, have been well carried out. The scope of the Journal has been enlarged, and its circle of influence greatly increased. We still continue the magnificent offer of Dr. Stowell's work on the *Microscopic Structure of a Human Tooth*, which is published at the moderate price of six dollars each. We have made such arrangements with the publishers that we can furnish it in connection with a subscription to **The Independent Practitioner for One Dollar and Fifty Cents. That is, to every one who will send in Four Dollars we will send the Independent Practitioner one year and a copy of this beautiful and useful atlas.**

This offer is intended only for subscribers in America. Foreign residents within the postal union must enclose fifty cents extra for prepayment of postage upon the Journal. Our regular rate of subscription to such is three dollars per annum.

As the book absolutely costs more than the sum for which it will be furnished, the money must accompany the subscription. Nor will the book be sold separately. It will only be sent in connection with a subscription to this journal.

The book will be sent either by mail or express. If it is desired that it should be sent by mail, the postage (twenty-five cents) should be sent. All remittances, for the present, should be sent to the Editor, Dr. W. X. Sudduth, care of Dr. W. C. Barrett, No. 208 Franklin Street, Buffalo, N. Y. Postal Orders, Postal Notes, or New York Drafts are the safest and most convenient.

With this number THE INDEPENDENT PRACTITIONER has been transferred to a new and enlarged syndicate—no material change is, however, to be made in the form of the journal until the close of the volume. We hope to hold all our old subscribers, and obtain many new ones. You may hand your subscription to any one of the stockholders, a list of whose names is here appended.

GEO. S. ALLAN
R. R. ANDREWS
H. A. BAKER
W. C. BARRETT
A. P. BEALE
S. T. BEALE
C. S. BECK
G. V. BLACK
E. A. BOGUE
C. F. W. BODECKER
W. G. A. BONWILL
EDWARD C. BRIGGS
THOS. BRADLEY
ALBERT H. BROCKWAY
WM. CARR
DWIGHT M. CLAPP
JOHN D. CODMAN
CHAS. D. COOK
EDW. T. DARBY
D. M. DAVIDSON
WM. H. DWINELLE
CHAS. J. ESSIG
J. N. FARRAR
T. FILLEBROWN
W. B. FINNEY
T. A. FLETCHER
M. W. FOSTER
CHAS. E. FRANCIS
W. F. FUNDENBERG
W. H. FUNDENBERG
E. C. FUNDENBERG
E. S. GAYLORD
J. P. GERAN
S. H. GUILFORD
JOHN J. HART
O. E. HILL
J. F. P. HODSON
J. MORGAN HOWE
ROB'T HUEY
LOUIS JACK
G. L. S. JAMESON
C. A. KINGSBURY
EDW. C. KIRK
W. T. LA ROCHE
W. K. LEECH
WILBUR F. LITCH
J. BOND LITTIG
BENJAMIN LORD
GEO. W. LOVEJOY (Canada)
JAMES McMANUS
E. V. McLEOD
DAN'L NEAL McQUILLAN
HORATIO C. MERIAM
W. D. MILLER (Berlin)
W. E. PAGE
S. B. PALMER
C. B. PARKER
S. G. PERRY
C. N. PEIRCE
CHAS. E. PIKE
W. A. PHREANER
W. E. PINKHAM
H. C. REGISTER
L. D. SHEPARD
EUGENE SMITH
B. HOLLY SMITH
S. G. STEVENS
W. X. SUDDUTH
AMBLER TEES
J. D. THOMAS
JAMES TRUMAN
WM. H. TRUEMAN
F. F. VAN WOERT
W. W. WALKER
I. T. WARDWELL
C. S. WARDWELL
G. W. WARREN
L. S. WATERS
GEO. W. WELD
JULIUS G. W. WERNER
JACOB L. WILLIAMS
R. B. WINDER
C. A. WOODWARD
J. A. WOODWARD

